# Adropin as A Fat-Burning Hormone with Multiple Functions—Review of a Decade of Research

**DOI:** 10.3390/molecules25030549

**Published:** 2020-01-27

**Authors:** Mariami Jasaszwili, Maria Billert, Mathias Z. Strowski, Krzysztof W. Nowak, Marek Skrzypski

**Affiliations:** 1 Department of Animal Physiology and Biochemistry, Poznań University of Life Sciences, 60-637 Poznań, Poland; mjasaszwili@gmail.com (M.J.); maria.billert@gmail.com (M.B.); kwnowak@up.poznan.pl (K.W.N.); 2 Department of Hepatology and Gastroenterology, Charité-University Medicine Berlin, D-13353 Berlin, Germany; mathias.strowski@charite.de; 3 Department of Internal Medicine-Gastroenterology, Park-Klinik Weissensee, D-13086 Berlin, Germany

**Keywords:** adropin, *Enho*, adiposity, metabolism, type 2 diabetes, liver, cancer, cardiovascular system

## Abstract

Adropin is a unique hormone encoded by the energy homeostasis-associated (*Enho*) gene. Adropin is produced in the liver and brain, and also in peripheral tissues such as in the heart and gastrointestinal tract. Furthermore, adropin is present in the circulatory system. A decade after its discovery, there is evidence that adropin may contribute to body weight regulation, glucose and lipid homeostasis, and cardiovascular system functions. In this review, we summarize and discuss the physiological, metabolic, and pathophysiological factors regulating *Enho* as well as adropin. Furthermore, we review the literature addressing the role of adropin in adiposity and type 2 diabetes. Finally, we elaborate on the role of adropin in the context of the cardiovascular system, liver diseases, and cancer.

## 1. Discovery of Adropin and the Adropin Receptor

In 2008, Kumar et al. identified a new hormone called adropin [1]. The authors of this pioneer work studied expression of genes in C57BL/6J (B6) melanocortin-3 receptor-deficient (Mc3r−/−) mice, which allowed for identification of an unknown liver transcript downregulated in obesity. Using bioinformatics and molecular biology it was found that this transcript encodes a secreted protein that was termed adropin (this name originated from Latin “aduro” which means “to set fire to” and “pinquis” which means “fats or oils” [1]). Secreted adropin protein is composed of 43 amino acids, and is produced by proteolytic cleavage of 76 amino acid precursors. Notably, the amino acid sequence of adropin is highly conserved among the species and is identical in rat, mouse, human, and pig [1]. Unfortunately, the plasma half-life of adropin is still unknown and remains to be determined. 

Adropin is encoded by the energy homeostasis-associated gene (*Enho*), which is expressed mainly in the brain and the liver. However, it is also detected in peripheral tissues such as in the heart, lung, kidney medulla, muscles, peripheral blood mononuclear cells, and breast cancer cells [1,2,3]. Furthermore, adropin protein is present in the circulatory system of animals and humans [1,4]. There are two studies indicating that biological effects of adropin are mediated via direct interaction with the G protein-coupled receptor—GPR19 receptor. Stein et al. found that in rats, adropin suppresses water intake via activation of GPR19 in the brain [5]. Moreover, it was found that adropin modulates E-cadherin expression in breast cancer cells via activation of GPR19 [6]. In addition, another study reported that adropin modulates pyruvate dehydrogenase in cardiac cells in a GPR19-dependent manner [7]. However, it should be pointed out that a recent study failed to confirm an interaction of GPR19 receptors with adropin [8]. Nevertheless, it must be noticed that there is evidence indicating that adropin is a plasma membrane protein modulating physical activity and motor coordination via NB-3/Notch signaling in the brain [9]. Thus, adropin may represent a protein with multiple functions, acting as a secreted factor and/or membrane protein.

Ten years after adropin’s discovery there is emerging evidence indicating that this peptide is involved in controlling metabolism and energy homeostasis. In this review we discuss regulation of *Enho* mRNA expression, adropin production by nutritional factors, and metabolic abnormalities and diseases. Moreover, we summarize findings indicating that adropin modulates glucose and lipid metabolism in obesity and type 2 diabetes mellitus. Furthermore, we elaborate on the role of adropin in the cardiovascular system, cancer, reproduction, and liver diseases.

## 2. Regulation of *Enho* mRNA Expression 

As mentioned above, obesity caused by melanocortin receptor or leptin deficiency leads to reduced *Enho* mRNA expression in the liver [1]. Interestingly, caloric restriction in melanocortin receptor-deficient mice normalized *Enho* mRNA expression in the liver. Furthermore, *Enho* mRNA expression is affected by the composition of diet. Mice fed a high-fat diet (60% kJ from fat) for 3 months had reduced *Enho* mRNA in the liver as compared with lean controls [1]. Suppression of hepatic mRNA expression was also reported in *Enho* mice fed a high-fat diet for 31 days [10]. In contrast, exposure to a high-fat diet for 2 days resulted in an increase of hepatic *Enho* mRNA expression. A similar effect was observed after 28 days of feeding with a high-fat diet. Moreover, *Enho* mRNA expression in the liver is downregulated after 10 days of fasting [1]. To further elucidate the mechanism by which *Enho* mRNA expression is upregulated by diet enriched in fat, Kumar et al. studied the potential role of intracellular lipid sensors in this process. Surprisingly, in hepatocarcinoma HepG2 cells’ *Enho* mRNA expression was downregulated in response to treatment with different nuclear liver X receptor (LXR) agonists (GW3965 or TO9). Of note, LXR controls cholesterol and triglyceride metabolism. Contribution of LXRα in downregulation of *Enho* mRNA expression was confirmed in vivo. In mice, treatment with LXRα agonist GW3965 was accompanied by reduction of *Enho* mRNA expression in the liver [1]. Thus, rapid upregulation of *Enho* mRNA expression by lipids is not mediated via LXR activation. However, a recent work evaluating the rhythmicity of *Enho* mRNA expression [10] found that *Enho* mRNA expression is mediated via the nuclear receptors RORα and RORγ. Noteworthy, the same study showed that liver *Enho* mRNA expression is downregulated by cholesterol in vitro [10]. It is worth noting that an in vivo study showed that the expression pattern of *Enho* displays rhythmicity [2]. A recent study found that in the liver and majority of central and peripheral tissues in Rhesus macaques, *Enho* mRNA is mainly expressed during the day time [2]. In summary, these results indicate that *Enho* mRNA is downregulated by fasting, while its modulation by a high-fat diet appears to be biphasic. Short exposure to a diet enriched in fat (up to 1 month) causes stimulation of liver *Enho* mRNA while hepatic *Enho* mRNA decreases in animals challenged with a high fat diet for 2 months.

## 3. Modulation of Adropin by Body Mass Index (BMI), Diet, and Diabetes

Several studies consistently shown that adropin levels in serum are affected by diet and depend upon metabolic diseases. Serum adropin levels are upregulated in mice fed a high-fat diet for 48 h [11]. In contrast, in mice with high-fat diet-induced obesity, serum adropin levels are low (<1 ng/mL). An inverse correlation of adropin levels and body mass index (BMI) was also confirmed by human studies [4,12,13,14,15,16,17], suggesting that a low level of adropin is a hallmark of obesity. Nevertheless, a recent detailed study showed that this association should be interpreted cautiously. For example, it was shown that in young lean men adropin levels are increased [10]. However, the same study showed that increased circulating levels of adropin is a risk factor for obesity in the middle and late stages of life [10]. In addition, serum adropin levels are also affected by sex. For example, women have lower circulating adropin levels as compared to men [4]. Furthermore, it was shown that in men, but not in women, circulating adropin is negatively associated with low-density lipoprotein (LDL) cholesterol levels [10].

This is in line with the results of in vivo and in vitro studies that showed that cholesterol suppresses *Enho* mRNA expression leading to lower adropin production. However, adropin overexpressing mice are not protected from hypercholesterolemia and atherosclerosis [10]. Therefore, adropin is not involved in cholesterol uptake from nutrition or cholesterol biosynthesis. In addition to lipids, it was shown that circulating adropin levels can be affected by carbohydrates intake. For example, a fall of adropin in serum was detected in mice fed a high-carbohydrate diet [11]. Interestingly, it was shown that glucose consumption suppresses adropin levels in circulation while fructose supplementation has an opposite effect [18]. Importantly, stimulation of adropin by fructose was more pronounced in humans with higher triacylglycerol levels, suggesting a role of lipids in regulating circulating adropin. Recently, it was shown that low levels of circulating adropin can be used as a predictor of body weight gain and metabolic dysregulation in Rhesus macaques challenged with a high sugar (fructose) diet [2]. Animals with low adropin levels had higher fasting glucose as well as leptin levels in response to a fructose challenge. Additionally, the same study showed an inverse correlation between serum adropin levels and apolipoprotein C3 in animals fed a high-fructose diet [2]. It is worth noting that high levels of apolipoprotein C3 are positively correlated with plasma triacylglycerol [19]. Consistently, increased levels of apolipoprotein C3 in animals with low levels of adropin, which were fed a high-fructose diet, were accompanied by more severe hyperglycemia [2].

There is growing evidence demonstrating that circulating adropin levels depend upon diet preferences. It was shown that in women but not in men, serum adropin concentration positively correlated with fat intake [20]. In addition, humans with low adropin levels consume more carbohydrates (simple and complex carbohydrates) [21]. Nevertheless, it is unknown whether diet affects adropin levels or whether adropin influences nutritional habits. Therefore, an association of adropin with diet preferences needs to be discussed cautiously.

In summary, adropin levels are affected by body weight, diet composition, and various diet preferences. Importantly, low levels of adropin are predictors of body weight gain in animals challenged with a fructose-enriched diet.

There is evidence indicating that adropin levels can be affected by diabetes. Serum levels of adropin are lower in type 2 diabetic patients as compared to healthy controls [22]. In addition, low levels of adropin in type 2 diabetes mellitus are a risk factor for endothelial dysfunction [23]. Downregulation of adropin in circulation in type 2 diabetes was also reported by Chen et al. [24]. These patients, who also have fatty pancreases, have lower serum levels of adropin [24]. It is also important to note that patients with lower adropin levels show a Cys56Trp mutation in the *Enho* gene [24]. Lower levels of adropin in circulation was also reported in women with gestational diabetes [25,26]. By contrast, serum levels of adropin in type 2 diabetic patients were high [27]. Aydin et al. studied the effects of type 1 diabetes induction on adropin levels in rats. This study found that STZ-treated type 1 diabetic rats have higher adropin levels in serum compared with that of the control group [28]. Moreover, this study demonstrated that adropin concentration was increased in pancreas, liver, kidney, brain, and cerebellum. Similarly, another study showed increased levels of adropin in kidneys and muscles in type 1 diabetic rats [29]. In contrast, a recent work found that adropin levels are downregulated in children who suffer from type 1 diabetes [30]. Therefore, studies testing the concentration of adropin in both types of diabetes provided partially contradictory data. It cannot be excluded that species differences (rats vs. humans), progress of disease, and age of patients (children vs. adults) could account to these differences.

Furthermore, similar to *Enho* mRNA expression, circulating adropin levels are affected by diet composition. Glucose suppresses serum adropin levels, while fructose has the opposite effect. Nevertheless, the mechanism by which glucose and fructose differently modulate adropin levels remains to be investigated.

## 4. The Role of Adropin in Controlling Adiposity and Lipid and Glucose Metabolism

Studies on genetically engineered animals provided strong evidence indicating that adropin contributes to the modulation of adiposity and metabolism of glucolipids. It was found that adropin-overexpressing mice are protected from body weight gain when fed a high-fat diet for 6 or 8 weeks [1]. Of note, attenuation of body weight gain was accompanied by reduction of fat mass. On the other hand, the same study showed that adropin-overexpressing transgenic mice (Adr-Tg) challenged with a high-fat diet for 3 months had similar body weight as compared to wild type animals. These results suggest that adropin overexpression delays but not completely prevents diet-induced body weight gain. Moreover, it was found that Adr-Tg mice (male and female) fed a high-fat diet have lower fasting levels of insulin and triglyceride. In contrast, blood glucose levels were not affected by adropin. In addition, the same study showed that adropin overexpression is associated with reduced insulin resistance (the homeostatic model assessment of insulin resistance—HOMA-IR) and improved glucose tolerance [1]. Overall, these results provide strong evidence that adropin overproduction improves insulin sensitivity and glucolipid metabolism in obesity. This statement is supported by the results of studies in animals treated with exogenous adropin. Kumar et al. found that obese mice treated with adropin eat less and lose body weight. In addition, less striking hyperinsulinemia as well as attenuated hepatic steatosis are hallmarks in these mice [1]. Moreover, adropin-treated mice have low expression of lipogenic genes in the liver. This relationship was confirmed in animals treated with adropin for two weeks [1]. In addition to the beneficial metabolic effects in diet-induced obesity, adropin reduces blood glucose level, improves insulin sensitivity, and suppresses inflammatory markers in a rat model of type 2 diabetes [31]. Overall, these results collectively show that adropin is able to attenuate metabolic abnormalities in obesity as well as type 2 diabetes. 

To elucidate the effects of adropin on metabolism, several studies characterized the direct effects of adropin on glucose and lipid metabolism. Importantly, it was found that adropin may contribute to the modulation of glucose synthesis. Thapa et al. reported low levels of basal and insulin-induced glucose production in the liver of diet-induced obesity (DIO) mice treated with adropin (450 nmol/kg b.w.) for three days [32]. Contribution of adropin to the hepatic glucose metabolism was also reported by Gao et al. [33]. This elegant in vivo work demonstrated that in mice with diet-induced obesity, exogenous adropin causes an increase of IRS1, IRS2, and AKT phosphorylation suggesting that adropin increases hepatic insulin sensitivity [33]. The same study showed that adropin-treated mice have reduced endoplasmic reticulum stress and JNK activity in the liver. Supporting the results of a previous study, it was found that adropin suppresses glucose production in hepatocytes that are mediated of cAMP/PKA [33]. This signaling pathway plays a prominent role in promoting glucose synthesis in the liver.

Furthermore, adropin controls the metabolism of glucose and lipids in skeletal muscles. Animals lacking adropin have increased fatty acid oxidation in muscles [34]. In contrast, exogenous injection into animals or overexpression of adropin stimulates glucose oxidation and reduces lipid oxidation in muscles. This process is mediated via PGC-1α. These results suggest that adropin may improve energy homeostasis by promoting glucose utilization in skeletal muscles [34]. Similarly, an independent study confirmed that adropin promotes glucose utilization in muscles, which was associated with increased activity of pyruvate dehydrogenase, indicating that adropin enhances glycolysis [35]. Moreover, the same study showed that adropin is able to improve mitochondrial functions leading to attenuation of incomplete fatty acids oxidation in muscles in mice with diet-induced obesity [35].

An additional target organ of adropin is the white adipose tissue. In a mouse model of obesity, adropin treatment suppressed lipogenic genes expression in adipose tissue [1]. Further evidence indicating that adropin may contribute to preadipocytes and adipocytes functions was published by Stein et al. who found that adropin the activates GPR19 receptor in 3T3-L1 cells [5]. Of note, these cells are able to differentiate into adipocytes, thus are commonly used as a cell model for studding adipogenesis and mature adipocytes functions [36]. Similarly, adropin stimulated proliferation of 3T3-L1 cells and rat primary preadipocytes in our own study [37]. These effects are mediated via ERK1/2 and AKT dependent mechanisms. In contrast, adropin suppresses differentiation of these cells into mature adipocytes [37]. These results provide evidence that adropin modulates energy homeostasis by interacting with white adipocytes. Overall, these results showed that adropin improves insulin sensitivity and glucose and lipid metabolism in obesity. Furthermore, adropin is able to improve insulin sensitivity and reduce hyperglycemia in animal models of type 2 diabetes. The regulation of metabolism by adropin appears to be dependent upon an influence of hepatic glucose synthesis and enhanced hepatic glucose oxidation. Furthermore, adropin may contribute to energy homeostasis by affecting lipogenic genes expression in adipose tissue while suppressing adipogenesis. Nevertheless, more in vivo experiments are required to further confirm all these data.

## 5. Adropin in the Cardiovascular System

Growing evidence suggests that adropin modulates functions of the cardiovascular system. Several studies investigated a relationship between adropin level and cardiovascular complications in humans. Potential contribution of adropin to function of endothelial cells was reported by Topus et al. [23]. This study reported that endothelial dysfunctions in patients with type 2 diabetes is paralleled by low levels of circulating adropin [23]. An association between low levels of adropin and cardiovascular disorders was also reported. Patients with cardiac syndrome X (CSX) have low circulating adropin, suggesting that low levels of adropin are risk factors for CSX [38]. Yu et al. found that reduction of adropin levels in patients with coronary artery disease predicts the incidence of acute myocardial infarction [13]. Additionally, low level of adropin in blood was found to be a coronary atherosclerosis predictor in both healthy and type 2 diabetic patients [22]. Low levels of adropin were also detected in patients with coronary heart diseases [39]. Interestingly, adropin levels in patients with coronary heart diseases are generally lower as compared to individuals who additionally are diagnosed as depressive. Thus, downregulation of adropin in depression may contribute to impaired energy homeostasis in this disease [39]. Finally, low levels of circulating adropin is the predictor of late saphenous vein graft occlusion [40], suggesting that adropin deficiency may contribute to the pathogenesis of saphenous vein graft disease.

Several studies assessed the relationship between adropin levels and blood pressure. Altincik et al. observed no correlation between blood pressure and adropin levels in obese children [41]. In contrast, another study reported lower levels of adropin in adults with hypertension [42]. Low levels of adropin in hypertensive patients were also reported by Gulen et al. [43]. Nevertheless, this work found that adropin levels in hypertensive patients do not correlate with acute hypertensive target organ damage [43]. Similarly, low levels of adropin were also reported in pregnant women with hypertension [44]. In contrast, others showed that hypertensive patients have high levels of adropin [45]. Furthermore, treatment of hypertensive patients with antihypertensive drugs such as valsartan and amlodipine leads to increased serum adropin levels [45]. In summary, low adropin levels in the circulatory system are downregulated in patients with cardiovascular diseases and are often predictors of cardiovascular diseases-associated late complications.

A potential role of adropin in regulating the cardiovascular system is derived from in vitro and in vivo experiments. Lovren et al. showed that adropin is involved in controlling the functions of endothelial cells [46]. This study found that adropin is able to promote replication and migration of human umbilical vein endothelial cells (HUVECs) [46]. In addition, adropin-stimulated HUVEC cells form capillary-like tubes and have an improved endothelial barrier [46]. Furthermore, adropin protects endothelial cells from TNF-α-induced apoptotic death. These effects are mediated via AKT, ERK1/2, as well as eNOS kinases. Of note, beneficial effects of adropin detected in vitro were confirmed by an in vivo study showing that adropin stimulates neovascularization via activation of eNOS kinase [46]. Concordantly, another study revealed that adropin has the ability to suppress THP1 monocyte adhesion to HUVEC cells induced by TNFα, suggesting an anti-inflammatory potential of adropin [47]. Moreover, in apolipoprotein E (Apoe) deficient mice (animal model of atherosclerosis [48]) synthetic adropin led to attenuation of atherosclerotic lesions and monocyte/macrophage infiltration in the aorta [47]. In the context of the cardiovascular system, adropin may modulate the patency of the blood–brain barrier [49]. Yang et al. showed that exogenous adropin causes attenuation of endothelial cell permeability during ischemia, an effect mediated via the ROCK-MLC2 signaling pathway [49]. In a recent study, Wu et al. found that adropin protects against cardiomyocyte injury by ischemia/reperfusion [50]. Furthermore, adropin promotes glucose oxidation in mice a fed high-fat diet [51]. Similarly, it was found that adropin enhances insulin signaling and promotes glucose oxidation in mice fed a high-fat diet, which is associated with higher cardiac output [52]. Adropin levels are high after the aerobic exercise-training in humans [53]. Moreover, increased levels of adropin are associated with elevated NOx levels and reduced arterial stiffness. Thus, it is possible that adropin may promote arterial stiffness via NO-dependent signaling [53]. In line with this data, physical exercise upregulates circulating adropin in humans, which is accompanied by improved endothelial function [54]. Overall, these results suggest that low levels of adropin are associated with cardiovascular complication, such as endothelial dysfunction, cardiac syndrome X, or hypertension. Furthermore, adropin may be helpful in improving the functions of endothelial cells and cardiac metabolism. Moreover, adropin may be a link between exercise and improved cardiovascular system function in patients with cardiovascular diseases. Therefore, adropin is an interesting candidate that justifies further investigation in the context of therapeutic intervention in cardiovascular diseases.

## 6. Adropin in the Reproductive System

Expression of *Enho* and the role of adropin in male and female reproductive systems is not well characterized so far. In polycystic ovarian syndrome (PCOS), adropin levels are low [55,56,57,58]. However, it is unknown whether adropin deficiency is associated with PCOS development or if it is a consequence of metabolic and endocrine disturbances associated with PCOS. It is worth noting that downregulation of circulating adropin levels was detected in woman with normal and increased BMI [56,57]. Therefore, lower levels of adropin in PCOS patients is not caused by changes in body mass. However, it was found that adropin levels in woman with PCOS are associated with increased levels of TNF-α [56]. Since in PCOS patients TNF-α was found to modulate the production of hormones involved in energy hemostasis, such as adiponectin [59], it was suggested that lower levels of adropin in PCOS patients may be caused by increased TNF-α [56].

When discussing the role of adropin in reproduction, it is worth pointing out that adropin may be involved in fetal development. Adropin levels in cord blood are inversely correlated with birth weight [26]. By contrast, others failed to find any correlation between serum adropin concentration and birth weight [60]. However, in the first study, adropin levels were assessed in pregnant women with gestational diabetes, while the later work enrolled healthy women, only. These differences may explain discrepant observations.

Furthermore, studies on healthy women showed a positive correlation between adropin levels in cord blood and gestational age at birth and placental weight [60]. It is also important to note that adropin is present in human colostrum and milk [61] as well as in cow milk [62]. Therefore, since milk peptide hormones are implicated in the development of the gastrointestinal tract in neonates [63], it cannot be excluded that adropin contributes to postnatal gastrointestinal tract development in newborns.

Overall, these results suggest that adropin plays a role in the pathogenesis of PCOS. Furthermore, adropin in cord blood correlates with gestational age. Nevertheless, taking into account a limited number of studies so far, the impact of adropin on the reproductive system requires much more investigation.

## 7. Adropin and Cancer

Several studies found that adropin and its putative receptor GPR19 may be involved in the biology of cancer cells. *GPR19* mRNA is high in human breast carcinoma [6]. Furthermore, overexpression of *GPR19* in mesenchymal-like breast cancer stimulates E-cadherin expression and promotes the epithelial-like phenotype via the MAPK/ERK1/2 pathway [6]. Therefore, GPR19 activation may promote carcinogenesis and metastases formation by promoting mesenchymal-to-epithelial cell transition. On the other hand, is was reported that adropin is able to suppress viability and induce apoptosis in the breast cancer MCF-7 cell line [64]. Thus, the role of adropin and its therapeutic potential in breast cancer is unclear and requires more experiments.

Adropin levels are low in patients with endometrium cancer [65]. However, the role of adropin in the pathophysiology of endometrium cancer remains unknown. In conclusion, knowledge regarding the role of adropin and its putative receptor is poor and more studies are needed to elucidate the role of adropin in the pathophysiology or prognosis of cancer.

## 8. Adropin in Liver Disease

Regulation of *Enho* mRNA expression in the liver and the effects of adropin on hepatic glucose production were discussed above. In addition, adropin may be involved in more aspects of liver physiology. Prystupa et al. reported that serum adropin levels are high in alcoholic liver cirrhosis patients. Furthermore, upregulation of adropin in serum was positively associated with the severity of alcoholic liver cirrhosis, based on the Child–Pugh score [66]. Chen et al. demonstrated that non-alcoholic induction of steatohepatitis causes a fall of adropin levels in serum [67]. Furthermore, mice with adropin deficiency have enhanced hepatic steatosis, fibrosis, as well as inflammation (non-alcoholic steatohepatitis—NASH) when fed methionine-choline deficient or western diets. In contrast, synthetic adropin attenuates NASH development in mice [67]. Notably, protective effects of adropin on hepatocytes injury were also confirmed in in vitro cultured hepatocytes. Investigation of molecular signaling conferring beneficial effects of adropin peptide against liver damage showed that adropin promotes Nfr2 transcription activity [67]. Downregulation of serum adropin levels was also reported in patients with non-alcoholic liver fatty disease [68]. In addition, a more detailed study of these patients showed that adropin downregulation was more pronounced in patients with insulin resistance [68]. In summary, these studies show that adropin level is downregulated in patients with NASH as well as fatty liver disease. In vitro and in vivo studies showed that adropin may improve liver damage, suggesting a potential role of adropin in the treatment of liver diseases. In contrast, liver cirrhosis induced by alcohol was accompanied by higher adropin levels than in healthy controls, which suggests that circulating adropin levels are differently modulated in liver diseases.

## 9. Concluding Remarks

**Figure 1:**
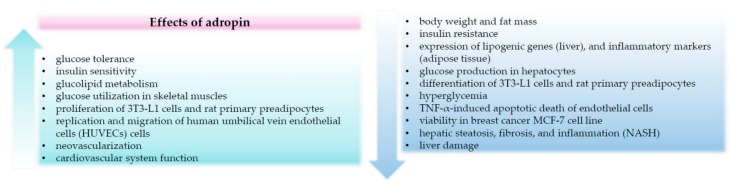
Summary of the biological effects of adropin.

In summary, more than ten years of studies on adropin have provided convincing evidence that this hormone modulates glucose and lipid metabolism. There is emerging evidence that adropin exerts beneficial metabolic effects in animal models of obesity. Adropin suppresses hepatic glucose production and improves insulin sensitivity in the liver. Furthermore, there is evidence that circulating adropin is downregulated in numerous cardiovascular diseases and may improve cardiovascular system function. Finally, adropin is able to improve liver function by protecting against hepatocytes injury. Unfortunately, as discussed above, some studies addressing the physiological and pathophysiological role of adropin have provided contradictory results. It cannot be excluded that these inconsistencies resulted from different assays used to detect adropin concentration in circulation. It is worth noting that in the majority of studies focused on the evaluation of adropin levels in different diseases, the utilized adropin antibodies were not well validated. Furthermore, as already mentioned before, the half-life of adropin in the circulation remains unknown. Moreover, recent results questioned whether adropin binds to GPR19. We speculate that solving these questions will allow for the study of a potential therapeutic relevance of adropin and its receptor/receptors. The biological effects of adropin are demonstrated in Figure 1. In summary, studies on adropin functions collectively suggest that adropin and its putative receptor may be relevant in the context of diagnosis and therapies of obesity, cardiovascular, and liver diseases.
